# Contrasting patterns of genetic divergence in two sympatric pseudo-metallophytes: *Rumex acetosa * L. and *Commelina communis * L.

**DOI:** 10.1186/1471-2148-12-84

**Published:** 2012-06-13

**Authors:** M Ye, B Liao, JT Li, A Mengoni, M Hu, WC Luo, WS Shu

**Affiliations:** 1 School of Life Sciences and State Key Laboratory of Biocontrol, Sun Yat-sen University, Guangzhou, 510275, People's Republic of China; 2 Department of Evolutionary Biology, University of Firenze, via Romana 17, I-50125, Florence, Italy; 3 Guangdong Provincial Academy of Environmental Science, Guangzhou, 510045, People's Republic of China

## Abstract

**Background:**

Patterns of genetic divergence between populations of facultative metallophytes have been investigated extensively. However, most previous investigations have focused on a single plant species making it unclear if genetic divergence shows common patterns or, conversely, is species-specific. The herbs *Rumex acetosa * L. and *Commelina communis * L. are two pseudo-metallophytes thriving in both normal and cupriferous soils along the middle and lower reaches of the Yangtze River in China. Their non-metallicolous and metallicolous populations are often sympatric thus providing an ideal opportunity for comparative estimation of genetic structures and divergence under the selective pressure derived from copper toxicity.

**Results:**

In the present study, patterns of genetic divergence of *R. acetosa * and *C. communis *, including metal tolerance, genetic structure and genetic relationships between populations, were investigated and compared using hydroponic experiments, AFLP, ISSR and chloroplast genetic markers. Our results show a significant reduction in genetic diversity in metallicolous populations of *C. communis * but not in *R. acetosa *. Moreover, genetic differentiation is less in *R. acetosa * than in *C. communis *, the latter species also shows a clustering of its metallicolous populations.

**Conclusions:**

We propose that the genetic divergences apparent in *R. acetosa * and *C. communis *, and the contrasting responses of the two species to copper contamination, might be attributed to the differences in their intrinsic physiological and ecological properties. No simple and generalised conclusions on genetic divergence in pseudo-metallophytes can thus be drawn.

## Background

Understanding the genetic basis of how organisms adapt to extreme and novel environments remains as one of the major challenges facing evolutionary biologists. Intraspecific differentiation of metallophytes is one of the most striking adaptive changes that result from natural outcrops of heavy metal-rich rock or mining activities [1-3]. Metallophytes refer to plant species that have developed metabolic mechanisms to resist, tolerate, or even thrive on toxic metalliferous soils [4-6]. Populations of metallophytes growing in metalliferous soils are often genetically distinct from those living in nearby non-metalliferous soils [7]. Metalliferous habitats are always characterized by their patchy distribution and therefore have been considered as ecological islands, which provide ideal opportunities to investigate the population differentiation in plants under severe edaphic pressure [8]. In the course of evolution, plants have adapted to widely differing metal availabilities in soils and therefore represent an important source of natural variation of metal homeostasis networks [9]. In the past 50 years, genetic differentiation within and among populations of metallophytes has attracted uninterrupted interest. Yet, the evolution of metallophytes remains heatedly debated [10-13]. Previous studies on genetic divergence of metallophytes have mainly focused on the following two aspects: (1) the effects of heavy metal pollution on genetic diversity, and (2) the origin and evolutionary history of metallicolous populations. In general, substantial founder and bottle-neck effects have been presumed to occur during the colonization of metallophytes in a heavy metal contaminated area. Therefore, a reduction in genetic diversity in metallicolous populations would be theoretically expected. This idea has been well-supported by several studies [14,15]. Conflicting results, however, have also been obtained in some cases where recently established tolerant populations maintain a high level of genetic variation comparable to that in non-metallicolous populations [16,17]. On the other hand, Schat *et al. *[18] have suggested that geographically distant conspecific metallicolous populations could have evolved independently and therefore constitute a polyphyletic group. The hypothesis of multiple and independent origins has been validated in metallicolous populations of several metallophytes such as *Noccaea caerulescens * (formerly known as *Thlaspi caerulescens *), *Silene paradoxa Arabidopsis halleri * and *A. lyrata *[17,19-21]. Beyond all doubt, both mosaic environments and edaphic conditions should contribute to the multiple origins of metallicolous populations. However, it is still not clear whether or not edaphic conditions played a more important role than geographical factors in this event. The conflicting results associated with such an issue might be due to: (1) differences in biological traits, ecological properties, population size, and history of individual populations of different species; and/or (2) various molecular markers (*i *.*e *. RAPD, RFLP, ISSR, and AFLP) employed in different studies. Moreover, it should also be noted that until now, most previous studies have focused on a single plant species only, which inevitably influences the comparability of results from different studies. Therefore, choosing two or more sympatric metallophytes with similar geographic distributions and population history, and then comparing their characteristics of genetic divergence might shed light on the evolutionary processes of plants under heavy metal pressure.

The copper (Cu) belts distributing along the middle and lower reaches of the Yangtze River are one of the largest areas of Cu mining in China. In this area, there are considerable spoil heaps and mineral deposits, some of which are currently in practice. A national ruin of an ancient Cu mine built over 1000 years ago (Da-Gong Mountain Mining Ruin) is located in this area, suggesting a long history of mining activities. Extensive Cu mining activities in this area have resulted in many waste heaps with elevated concentrations of Cu in soils, and the plant species growing in this area have experienced long-term selection under Cu toxicity derived from mining activities dating back to at least 1000 years. Although these waste heaps are often far away from each other, they harbor similar assemblages of metallophytes, which are distinctly different from the surrounding communities in non-metalliferous soils [22-24]. Both common sorrel *Rumex acetosa * L. (Polygonaceae) and Asiatic dayflower *Commelina communis * L. (Commelinaceae ) have consistently been documented as dominant plants on most of these cupriferous habitats and are also present in surrounding non-metalliferous sites [21,22]. Besides, *R. acetosa * is well-known metallophyte thriving on metalliferous soils of NW & SW Europe. Many edaphic ecotypes of *R. acetosa * from a wide range of soil types have been reported, some of which exhibit high potential for phytoremediation of contaminated soils [2,25,26].

*Rumex acetosa * is a perennial herb with an erect stem, about 40 to 120 cm high. Its flowering season ranges from March to May and the fruiting season ranges from April to June. Unisexual and dioecious flowers are arranged in acrogenous panicle inflorescences. Plants are capable of producing large numbers of small achenes encircled with an aliform membrane and therefore can be easily dispersed by wind. The species is commonly found along roadsides, in forests, and in habitats along rice fields in most regions of China [27]. In contrast, *C *. *communis * is an annual multi-branched herb with erect stems in the upper part and creeping stems in the lower part. It is able to grow clonally by elongation of the creeping stem, which bears a number of erect ascending shoots. It flowers from May to September and fructifies from June to November. Bisexual flowers are arranged in a cyme. *Commelina communis * often occupies habitats similar to those of *R. acetosa * and is mainly distributed in southeastern China [28].

Despite their similarities, the two pseudo-metallophtes *R. acetosa * and *C. communis * have different life histories (perennial vs annual) and reproductive systems (obligately outcrossing vs potentially selfing), which result in them being in different functional groups. Therefore, their metallicolous and non-metallicolous populations provide an ideal opportunity to study genetic divergence under selective pressure derived from Cu toxicity between taxa of two distinct functional groups.

Previous studies have shown that many factors such as life history and reproductive system may significantly influence population genetic divergence through their effects on genetic drift and gene flow [8,15-17]. We therefore hypothesized that the two sympatric pseudo-metallophytes *R. acetosa * and *C. communis * would differ greatly in population genetic divergence. To test our hypothesis, 12 *R. acetosa * populations and 13 *C. communis * populations collected from different sites along the Yangtze River of Eastern China were used to investigate comparative genetic differentiation patterns. AFLP (Amplified Fragment Length Polymorphism) and ISSR (Inter-Simple Sequence Repeats) were employed to assess genetic diversity and population structures. Six *R. acetosa * and 10 *C. communis * populations were selected to determine Cu tolerance of the metallicolous and non-metallicolous populations. In addition, two cpDNA regions (*psbJ-petA * and *3′rps16-5′trnK *) were sequenced and analyzed to infer uniparental patterns of colonization.

## Results

### Cu concentrations in soils

**Figure 1:**
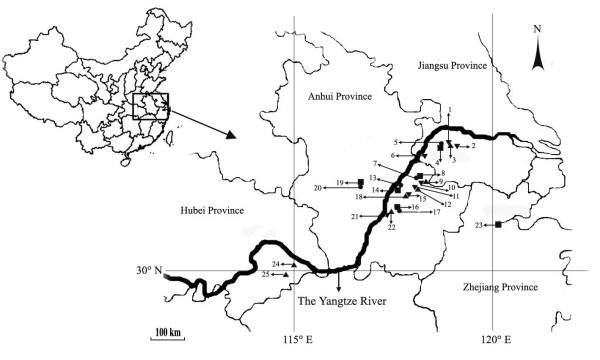
**The sampling locations of 12 *Rumex acetosa * and 13 *Commelina communis * populations**. · NM populations of *R. acetosa *; ▪ NM populations of *C. communis *; ▴ M populations of *R. acetosa *; ▾ M populations of *C. communis *.(1, 3: AJS; 2: JHS; 4, 5: JSCK; 6: TJ; 7, 8: DGSCK; 9, 10: DGS; 11, 12: FHS 13, 14: TLCK;15, 18: SZS; 16, 17: CZCK; 19, 20: SCCK; 21, 22: CZ; 23: HZCK; 24: TLS; 25: TSK).

Sampling locations for 12 *R. acetosa * and 13 *C. communis * populations were investigated at areas along the Yangtze River (Figure 1). The soil Cu concentrations recorded at these different sampling sites are shown in Table 1. In general, contaminated sites contained high concentrations of total and DTPA-extractable Cu, about 100 times greater than those in uncontaminated sites. Large differences in Cu concentrations were detected between cupriferous sites; the highest total and extractable Cu concentrations were 8587 and 2194 mg kg^−1^ at TLS, whilst the lowest values were 2787 and 359 mg kg^−1^ at FHS, respectively.

**Table 1:** **Locations of populations of *Rumex acetosa * and *Commelina communis * , and the total and extractable Cu concentrations in the substrates associated with plants sampled**

**Edaphic type**	**Latitude/Longitude**	**Populations (Abbreviation)**	***R. acetosa ***		***C. communis ***		**Concentration of Cu in substrates (mg kg**^**-1**^**, mean ± SD)**	
			***N ***_***i ***_**est.**	**Sample size**	***N ***_***i ***_**est.**	**Sample size**	**Total Cu**	**Extractable Cu**
M	30°52´N, 118°01´E	Fenghuangshan, Anhui (FHS)	> 500	13	> 1000	15	2787 ± 827 ^b**^	359 ± 85 ^b^
M	30°55´N, 117°53´E	Shizishan, Anhui (SZS)	100-500	11	500-1000	15	7966 ± 1197 ^a^	1778 ± 314 ^a^
M	30°56´N, 118°09´E	Dagongshan, Anhui (DGS)	> 500	15	500-1000	12	7033 ± 1819 ^a^	1769 ± 610 ^a^
M	30°26´N, 117°16´E	Tongshan, Anhui (CZ)	> 5000	15	> 5000	15	3941 ± 1228 ^b^	594 ± 279 ^b^
M	30°02´N, 115°01´E	Tonglvshan, Hubei (TLS)	n.a	n.a	> 1000	15	8587 ± 920 ^a^	2194 ± 219 ^a^
M	29°56´N, 114°53´E	Tongshankou, Hubei (TSK)	n.a.	n.a	< 500	13	4519 ± 988 ^b^	679 ± 142 ^b^
M	31°45´N, 118°33´E	Tongjing, Jiangsu (TJ)	< 100	11	n.a.	n.a	3895 ± 76 ^b^	927 ± 17 ^b^
M	32°06´N, 119°04´E	Anjishan, Jiangsu (AJS)	< 100	10	< 100	10	6572 ± 238 ^a^	1832 ± 75 ^a^
M	32°04´N, 119°05´E	Jiuhuashan, Jiangsu (JHS)	100-500	12	n.a.	n.a	3341 ± 100 ^b^	633 ± 20 ^b^
NM	30°56´N, 117°49´E	Tongling, Anhui (TLCK)	< 100	11	< 100	12	50 ± 0.3 ^c^	5.1 ± 0.3 ^c^
NM	31°12´N, 116°47´E	Shucheng, Anhui (SCCK)	100-500	13	100-500	14	3.7 ± 0.4 ^c^	2.0 ± 0.1 ^c^
NM	30°28´N, 117°17´E	Chizhou, Anhui (CZCK)	100-500	14	< 100	12	28 ± 18 ^c^	6.4 ± 0.2 ^c^
NM	30°57´N, 118°09´E	Nanling, Anhui (DGSCK)	< 100	11	100-500	15	28 ± 0.4 ^c^	7.2 ± 0.3 ^c^
NM	32°02´N, 118°55´E	Nanjing, Jiangsu (JSCK)	< 100	12	< 100	11	62 ± 28 ^c^	11 ± 0.3 ^c^
NM	30°15´N, 120°08´E	Hangzhou, Zhejiang (HZCK)	n.a.	n.a	100-500	12	23 ± 4.3 ^c^	6.4 ± 0.1 ^c^

*: M, metallicolous; NM, non-metallicolous; Ni est., approximative population size; n.a., data not available, the species is not present in the indicated site.

**: Data with different letters in the same column indicate a significant difference (*P * < 0.05, LSD test).

### Cu tolerance

**Figure 2:**
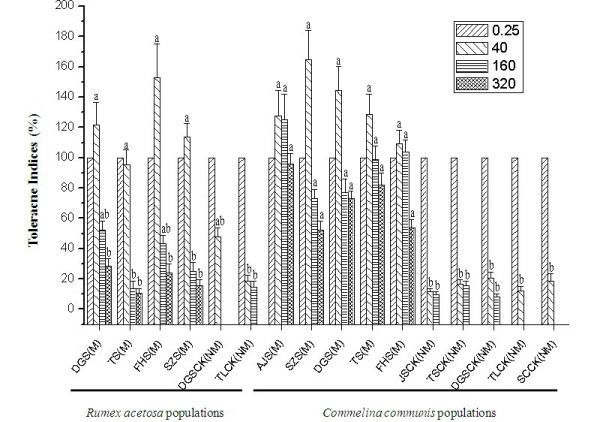
**Tolerance indices (%) based on relative root length of different populations of *Rumex acetosa * and *Commelina communis * under four Cu treatments in hydroculture (0.25, 40, 160, and 320 μmol L**^**-1**^**CuSO4, respectively; M: metallicolous; NM: non-metallicolous)**. Different letters on the error bars indicate a significant difference at *P * < 0.05 (LSD test) under the same treatment.

Tolerance indices based upon growth quotients of relative root length under the four Cu treatments for the different populations are shown in Figure 2. Overall, Cu tolerance indices for the 6 *R. acetosa * populations and 10 *C. communis * populations under four Cu treatments were significantly different between the metallicolous (M) and non-metallicolous (NM) populations; tolerance indices of M populations were significantly higher than those of NM populations for both species*. * Interestingly, tolerance indices of NM populations of both species were also similar. *Commelina communis * M populations exhibited higher tolerances than those of *R. acetosa * in the 160 μM and 320 μM Cu treatments. Shoot elongation was, with few exceptions, considerably depressed in all NM populations investigated when seedlings were treated with 40 μM Cu or above. Moreover, the treatment with 320 μM Cu which killed all NM plants revealed a higher tolerance in *C. communis * M populations than *R. acetosa * M populations (Additional file 1: Figure S1 and S2).

### Genetic diversity of *Rumex acetosa * and *Commelina communis * populations

The 6 primer combinations used in the AFLP analysis yielded 212 and 238 bands in total and the percentage of polymorphic loci was 84.4% and 86.5% for *R. acetosa * and *C. communis, * respectively. ISSR markers scored were 65 and 77 in total and the percentage of polymorphic loci was 66.2% and 74% for *R. acetosa * and *C. communis, * respectively. Nei’s gene diversity and Shannon indices of each population based on AFLP and ISSR data are given in Tables 2 and 3.

**Table 2:** **Genetic diversity statistics presented as Nei′s gene diversity (H) and Shannon index (H′) in *Rumex acetosa * and *Commelina communis * based on AFLP data (for each population separately and also divided in two types according to edaphic conditions)**

**Edaphic type**	**Populations**	**H**		**H′**	
		***R. acetosa ***	***C. communis ***	***R. acetosa ***	***C. communis ***
M	DGS	0.178	0.1536	0.258	0.222
M	FHS	0.143	0.1453	0.207	0.209
M	SZS	0.161	0.1429	0.233	0.206
M	CZ	0.181	0.1608	0.261	0.232
M	AJS	0.185	0.1244	0.267	0.179
M	JHS	0.185	n.a.	0.266	n.a.
M	TJ	0.196	n.a.	0.280	n.a.
M	TLS	n.a.	0.1464	n.a.	0.210
M	TSK	n.a.	0.1305	n.a.	0.188
	Mean ± SD	0.175 ± 0.018 a	0.143 ± 0.013 b	0.253 ± 0.025 a	0.206 ± 0.018 b
N	DGSCK	0.1777	0.231	0.256	0.332
N	TLCK	0.1731	0.229	0.249	0.331
N	CZCK	0.1812	0.230	0.260	0.331
N	SCCK	0.1541	0.232	0.221	0.334
N	JSCK	0.1885	0.235	0.272	0.339
N	HZCK	n.a.	0.222	n.a.	0.320
	Mean ± SD	0.175 ± 0.023 a	0.23 ± 0.005 a	0.252 ± 0.019 a	0.331 ± 0.006 a

*: M, metallicolous populations; N, non-metallicolous populations; n.a., data not available. Data followed by different letters in the same column indicate a significant difference (*P * < 0.001).

**Table 3:** **Genetic diversity statistics presented as Nei′s gene diversity (H) and Shannon index (H′) in *Rumex acetosa * and *Commelina communis * based on ISSR data (for each population separately and also divided in two types according to edaphic conditions)**

**Edaphic type**	**Populations**	**H**		**H′**	
		***R. acetosa ***	***C. communis ***	***R. acetosa ***	***C. communis ***
M	DGS	0.1532	0.1997	0.2476	0.3116
M	FHS	0.1641	0.1870	0.2610	0.2936
M	SZS	0.1616	0.1914	0.2553	0.2967
M	TS	0.1860	0.1958	0.2842	0.3060
M	AJS	0.1556	0.1646	0.2439	0.2558
M	JHS	0.1611	n.a.	0.2515	n.a.
M	TJ	0.1619	n.a.	0.2536	n.a.
M	TLS	n.a.	0.1538	n.a.	0.2446
M	TSK	n.a.	0.1528	n.a.	0.2391
	Mean ± SD	0.163 ± 0.011a	0.178 ± 0.020 b	0.257 ± 0.013a	0.278 ± 0.031 b
N	DGSCK	0.1543	0.2048	0.2472	0.3169
N	TLCK	0.1422	0.2016	0.2280	0.3108
N	TSCK	0.1813	0.2089	0.2776	0.3241
N	SCCK	0.1480	0.2584	0.2305	0.3899
N	JSCK	0.1603	0.2360	0.2464	0.3572
N	HZCK	n.a.	0.2558	n.a.	0.3830
	Mean ± SD	0.157 ± 0.015a	0.228 ± 0.026 a	0.246 ± 0.020a	0.347 ± 0.035 a

*: M, metallicolous; N, non-metallicolous; n.a., data not available.

Data followed by different letters in the same column indicate a significant difference (*P * < 0.05).

For *R. acetosa * no significant difference in Nei’s gene diversity between M populations and NM populations was detected. Further, there was no significant correlation between Nei’s gene diversity and the DTPA-extractable Cu concentrations in associated soils within each population (Pearson’s correlation coefficient r_S_ = 0.119, *P * = 0.712, and r_S_ = −0.045, *P * = 0.890, for AFLP data and ISSR data, respectively). In contrast, for *C. communis * significant (*P * < 0.001) differences in genetic diversity were detected between the M and NM populations, the latter having higher values than the former. Furthermore, significantly negative correlations between genetic diversity and DTPA-extractable Cu concentrations were detected (Pearson’s correlation coefficient r_S_ =–0.793, *P * = 0.001; r_S_ =–0.652, *P * = 0.016 for AFLP and ISSR, respectively).

### Genetic structure analysis

The genetic structure of *R. acetosa * and *C. communis * populations was investigated by an Analysis of Molecular Variance (AMOVA). Relatively high genetic differentiation among populations of both *C. communis *, (*F *_*ST *_ = 0.129, *P * < 0.0001) and *R. acetosa * (*F *_*ST *_ = 0.11, *P * < 0.0001) was detected from AFLP data. Conversely, for *R. acetosa * ISSRs were not able to detect significant differentiation among populations (*F *_*ST *_ = 0.003, *P * > 0.05), whilst a low differentiation was detected for *C. communis * (*F *_*ST *_ = 0.029, *P * < 0.0001). Mantel’s tests carried out to investigate the existence of any correlations between geographical and genetic distances (Additional file 2: Table S1) did not detect significant correlations for AFLP data in either plant species. In contrast, differentiation at ISSR loci did show significant correlation with geographical distance especially for *R. acetosa * (r = 0.88, *P * < 0.0001).

**Figure 3:**
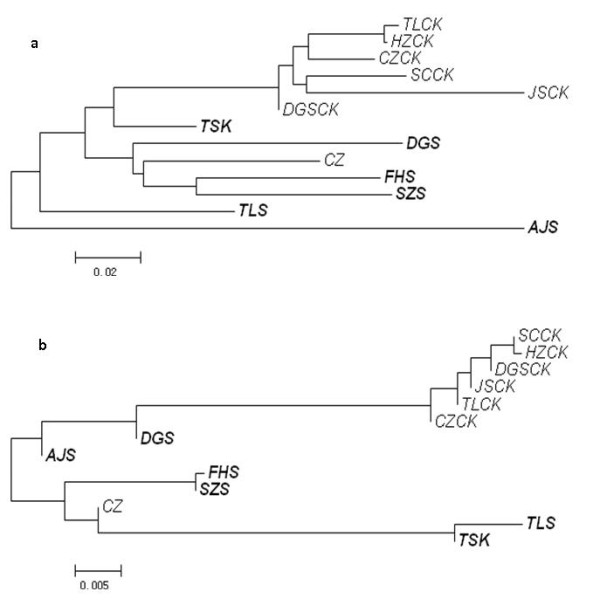
**NJ dendrogram of *C. communis * populations**. A, AFLP data; b ,ISSR data. In bold M populations. Scale bar, Slatkin’s linearized pairwise *F *_*ST *_.

**Figure 4:**
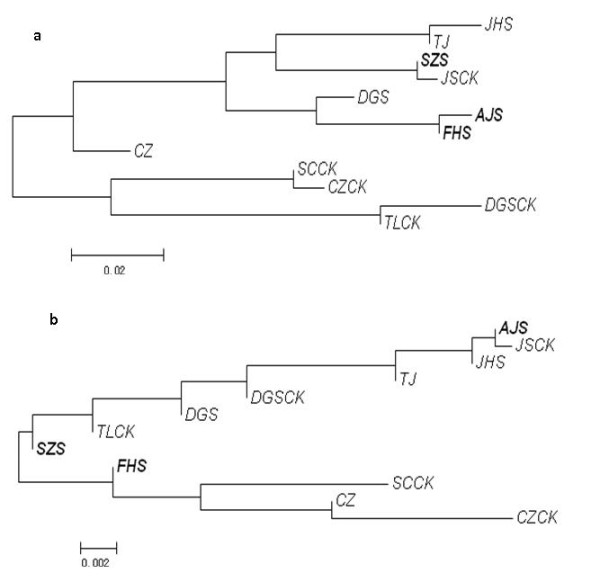
**NJ dendrogram of *R *  *. acetosa populations. * ** a, AFLP data; b ,ISSR data. In bold M populations. Scale bar, Slatkin’s linearized pairwise *F *_*ST *_.

Genetic relationships among populations were then investigated by computing NJ dendrograms from Slatkin’s linearized pairwise *F *_*ST *_ (Additional file 2: Table S2). Results are reported in Figures 3 and 4 for *C. communis * and *R. acetosa, * respectively. In general, only dendrograms based on AFLP were supported by significant P-values for pairwise *F *_*ST *_ for all pairwise comparisons. For *C. communis * (Figure 3), in both AFLP and ISSR data, M populations were more differentiated than NM populations, these latter being grouped in a single cluster (except for CZ population). Conversely, the *Rumex acetosa * populations were clustered into several groups which approximately reflected their geographic locations, supporting the results of the Mantel’s test. For example, the four populations sampled from Jiangsu Province (JSCK, TJ, AJS, and JHS) were clustered in one main branch in both AFLP and ISSR analyses (Figure 4).

Hierarchical partition was then computed at three levels (groups, populations and individuals). Groupings were related to both edaphic and geographic differences (Additional file 2: Tables S3 and S4 for *C. communis * and *R. acetosa, * respectively). Edaphic differentiation was strongly supported for *C. communis * (18.51% and 6.64% of total variance for ISSR and AFLP data respectively), whereas lower levels were found for *R. acetosa * (3.68 and 4.04% for ISSR and AFLP data). However, for *C. communis, * a geographical structuring of populations was also strongly supported by the ISSR data (11.01% of total variance).

### Chloroplast markers analysis

**Figure 5:**
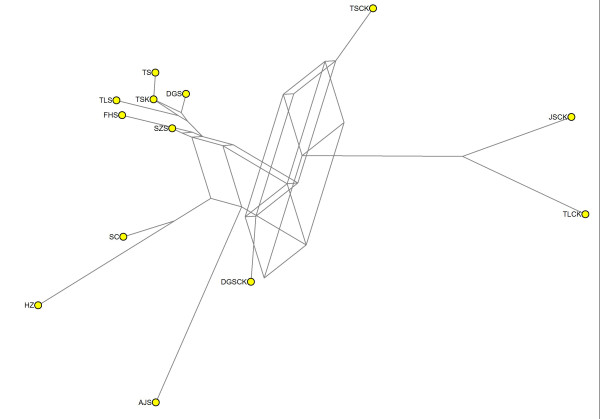
**Haplotype network of *Commelina communis * populations based on combined sequences of psbJ-petA and 3′rps16-5′trnK regions.**

Two cpDNA non-coding regions (*psbJ *-*petA * and *3′rps16-5′trnK *) of each population investigated were sequenced (GenBank accession numbers HM041054-HM041103, Additional file 2: Table S5). For *C. communis *, the aligned concatemers formed linearly combining the sequences of *psbJ-petA * and *3′rps16-5′trnK * regions resulted 1492 bp long. The number of variable sites was 60, 37 of which were parsimony-informative. For *R. acetosa, * the alignment was 1688 bp long, with 22 variable sites, only 3 of which were parsimony-informative. However, due to the low sequence variability, haplotype network was not informative on *R. acetosa *, while for *C. communis * populations some clustering of haplotypes was recognized (Figure 5). In particular for *C. communis *, four out of five metallicolous populations (TSK, CZ, TLS, SZS, DGS, and FHS) were included in one single group, suggesting a common chloroplastic (*i.e. * maternal) origin.

## Discussion

Divergence of metallophytes under heavy metal selection has been characterized by other researchers, with a focus on only a few species, notably *N. caerulescens *[29-33], *A. halleri *[1,16,34-36], *Cistus ladanifer *[37] and species in the genus *Silene *[15,18,33,38,39]. To our knowledge, the present study is the first report on a comparison of the genetic divergence of two sympatric metallophytes.

Heavy metal tolerance in higher plants is a well-documented example of micro-evolution and is a basic necessity for survival in metal-contaminated soils [9,40]. Based on the assumption that genotypes with a sufficiently high level of metal tolerance are rare in non-metallicolous populations, the founder effect was hypothesized to be a result of strong selection occurred during the colonization of contaminated areas by metallophytes [8]. A comparison of heavy metal tolerance among populations from metalliferous and non-metalliferous sites would provide an estimation of the levels of stress occurred during the colonization of heavy metal-rich areas. Differences in heavy metal tolerance among populations of metallophytes have been reported widely (see [41-44] for examples). In general, populations or ecotypes growing in contaminated soils exhibit higher levels of heavy metal tolerance than those from uncontaminated sites.

Tolerance index is a measure that can be used to assess the relative degree of tolerance of various plant varieties [45]. In this sense, it is important to test the Cu tolerance of non-tolerant species (or non-metallicolous populations in our case) as a control. On the other hand, in most of the relevant literature [4,45], heavy metal tolerances of non-metallicolous species (populations) have been compared to those of metallicolous ones. We therefore believe that this kind of comparison is suitable for a relative assessment within our two species. Yet, it should be noted that we cannot establish the presence of any baseline (constitutive) tolerance above that of other local species.

In the present study, significant differences in Cu tolerance at the population level were detected. Further, differences in Cu tolerance between the two species under investigation were also detected. Under the similar level of Cu stress, *C. communis * exhibited higher Cu tolerance than *R. acetosa *, especially when Cu concentration reached the maximum of 320 μmol L^−1^, Cu tolerances of the 4 *C. communis * populations from cupriferous sites were significantly higher than those of the 4 *R. acetosa * populations from the same sites (*P * < 0.05). Interestingly, there was no significant difference in Cu tolerance of non-metallicolous populations between the two species, suggesting a possible similar level of selective pressure on the ancestral non-tolerant colonizers of metalliferous sites. Although the concentrations of Cu in the contaminated sites we sampled were extremely high when compared with those of the non-metalliferous sites, distinct differences in Cu concentration were also observed among contaminated sites. Therefore, heterogeneity in the metalliferous sites could partly explain the difference in Cu tolerance among metallicolous populations. However, considering that the Cu concentrations in contaminated sites were generally 100 times greater than those in uncontaminated sites, the significant differences in Cu tolerance should be mostly attributed to the intense and long-term selection due to heavy metal stresses and to the differences in biological traits of the two metallophytes. Because they were sympatric in most sampling sites, and so subjected to the similar edaphic conditions. On the other hand, it is possible that the heterogeneous nature of the metalliferous sites could also partly explain the difference of Cu tolerance between the two species. Because there were some significant differences in Cu concentration among the metalliferous sites (Table 1). However, it should also be noted that we cannot exclude the potential contribution of maternal environmental effects on Cu tolerance of different populations of the two species, since field collected seeds were used and in the present study measurements were taken on young seedlings.

In order to determine more thoroughly whether the different phenotypes observed are linked to population histories and are reflected in the genetic diversity of populations, three different molecular techniques (AFLP, ISSR, and cpDNA) were employed to analyze the genetic structures and genetic relationships among different populations of the two metallophytes. Firstly, we found that levels of genetic diversity in the two co-occurring species were distinctly different. For *R. acetosa *, no significant difference in genetic diversity between metallicolous and non-metallicolous populations was found, whereas significantly lower levels of genetic diversity were detected in metallicolous populations than in non-metallicolous populations of *C. communis *. Similarly, Mengoni *et al. *[15] reported a significant decrease in genetic diversity of populations in *S. paradoxa * collected from Cu deposits. However, contrasting results were obtained in studies on other species. For example, the metallicolous and non-metallicolous populations of *A. halleri *[16], *N. caerulescens *[31] and *Onosma echioides *[46] maintained similar levels of genetic diversity. In a more recent study on *N. caerulescens *, genetic differentiation linked to heavy metal concentrations in soil was detected, and the gene flow observed at some nuclear loci was shown to be significantly reduced between plants encountering different levels of heavy metal contamination in the soil, suggesting that natural selection limits gene flow between metalliferous and non- metalliferous locations [32]. In the present study, the reduction of genetic diversity in metallicolous populations of *C. communis * might be due to a strong selective pressure during the colonization of Cu-contaminated sites by this species, coupled with a limited gene flow from surrounding non-metallicolous populations (as suggested by the relatively high values of pairwise *F *_*ST *_). In contrast, the similar levels of genetic diversity in metallicolous and non-metallicolous populations of *R. acetosa * might be due to substantial gene flow between populations, as indicated by the low values of pairwise *F *_*ST *_*. * High levels of gene flow between metallicolous and non-metallicolous populations of metallophytes have previously been reported in some other comparable studies [16,17,31]. In addition, non-metallicolous populations of *R. acetosa * showed higher levels of background tolerance to Cu (especially for DGSCK) compared to those of *C. communis. * Considering that metallicolous populations are presumed to evolve from non-metallicolous populations, this evidence may imply that selection for Cu tolerance in *C. communis * has been greater than in *R. acetosa *, although present-day levels of Cu tolerance in metallicolous populations are lower in *R. acetosa *.

Concerning that the habitats of metallicolous populations of metallophytes are often fragmented and disjunct, it is unlikely that dispersal from a single tolerant ancestral population could have produced the wide ranges of geographic distribution today. Hence, the hypothesis of polyphyletic origin is proposed and has been subsequently corroborated by considerable data [15,17-19,31]. In the case of *R. acetosa *, metallicolous populations branched in separate positions in the NJ dendrograms obtained by AFLP and ISSR data, supporting the hypothesis of independent origins from nearby normal populations. Furthermore, we also found that there was a direct correlation between genetic differentiation and geographical distances between pairs of *R. acetosa * populations (in particular for ISSR markers, Additional file 2: Table S1). For *C. communis *, we also speculate that the metallicolous populations evolved independently, since the site-to-site distances of the metallicolous populations were too far to be overcome by natural dispersal. However, metallicolous and non-metallicolous populations of *C. communis * had a distinct branching pattern in the NJ dendrograms with AFLP and ISSR data. The non-metallicolous populations were included in one group, whilst the metallicolous populations dispersed into single long branches. Additionally, components of variance of differentiation between populations of *C. communis * were also higher than those of *R. acetosa *. These results suggested a low gene flow in *C. communis * not only between metallicolous and non-metallicolous populations, but also among metallicolous populations, supporting higher levels (and possibly rates) of genetic differentiation.

The contrasting pattern of genetic differentiation between *C. communis * and *R. acetosa * was also confirmed by the analysis of chloroplast markers. Though this analysis was performed on only one individual per population, results for *C. communis * were similar with those obtained with AFLP, suggesting the presence of one main cluster of metallicolous populations. On this basis, it could be reasonable to hypothesize that metallicolous populations of *C. communis * may derive from seeds of one single ancestral population. Interestingly, we observed a very low variability of chloroplast sequences of *R. acetosa *, which seemed to be consistent with the idea of a recent spread and low differentiation between metallicolous and non-metallicolous populations.

The contrasting results of the two metallophytes also implied that populations of *C. communis * were more remarkably influenced by heavy metal stress than those of *R. acetosa *, although the genetic structures of the two species were influenced by geographic isolation and heavy metal contamination.

Although ecogeographic isolation has long been viewed as the most important reproductive barrier in plants [47], barriers to gene flow were also demonstrated between non-metallicolous and closely adjacent metallicolous populations of some species such as the grass *Anthoxanthum odoratum *[48]. Additionally, it has been reported that heavy metal contamination could have a greater impact on the population structure of the hyperaccumulator *Sedum alfredii * than geographic distance [49]. Based on our results, we can therefore speculate that both heavy metals and geographic distance play a significant role in determining the population structure of *R. acetosa *. In contrast, Cu contamination seemed to play a more important role in determining the population structure of *C. communis * than geographic distance.

In previous studies, it has been shown that the genetic divergence and evolutionary processes of plants could be affected by various factors, such as edaphic conditions, stress intensity and duration, geographic isolation, bottleneck and founder effects, life history, reproductive system, and so on [16,29]. Considering that *R. acetosa * and *C. communis * populations in the present study almost share the same edaphic conditions (mainly Cu toxicity) and geographic distribution pattern, the different characteristics of genetic divergence might result largely from their different life history and reproductive system. On the one hand, as a perennial, unisexual, and dioecious species, *R. acetosa * may have more opportunities to experience gene exchange among individuals and gene flow between populations than the bisexual annual plant *C. communis *. In addition, the winged seeds of *R. acetosa * are presumed to have higher dispersal capacity than those of *C. communis *, which might enhance the success of colonization by *R. acetosa *[27,28].

## Conclusions

Contrasting patterns of genetic divergence in two pseudo-metallophytes that experience similar selective pressures provides insight into evolutionary processes under heavy metal stress. Our findings indicate that genetic divergence of metallophytes is the result of the interactions between biological properties of a species (*e.g. * seed dispersal, background tolerance) and geographical patterns of colonization. Yet, no simplistic conclusions (*e.g. * the presence of founder effects *etc. *) can be drawn concerning the microevolutionary dynamics of all metallophytes.

## Methods

### Sampling sites

Nine metalliferous sites along the Yangtze River of Eastern China and six nearby non-metalliferous sites were selected as sampling sites (Table 1 and Figure 1). The sampling was conducted during the period from May to June 2007.

### Soil sampling and analysis

From 3 to 10 soil samples were collected at each sampling site (0–20 cm depth). Soil samples were air-dried and passed through a 2-mm sieve. For analysis of total Cu, soil samples were digested with 65% HNO_3_ + concentrated HClO_4_ (5:1) [50]; for analysis of extractable Cu, soil samples were extracted with a diethylenetetraminepentaacetic acid (DTPA) solution [51]. Cu concentrations in digested or extracted solutions were then determined by atomic absorption spectrometry (Hitachi-Z-5300, Hitachi, Japan).

### Hydroponic experiment

Six *R. acetosa * populations and 10 *C. communis * populations were selected for hydoponic experiments to investigate Cu tolerance in each population (Figure 2). A bulk collection of mature seeds was made from 5–10 plants sampled in the field, depending on the number of seeds on each plant. Seeds were germinated on sand and then 3-week old seedlings were transferred to a nutrient solution in a glasshouse equipped with supplementary lighting with fluorescent tubes (44 W/m^2^; 14-h photoperiod; 24–26°C). The composition of the nutrient solution was as follows: Ca(NO_3_)_2_·4H_2_O 2.00 mmol L^−1^, KH_2_PO_4_ 0.10 mmol L^−1^,MgSO_4_·7H_2_O 0.50 mmol L^−1^,KCl 0.10 mmol L^−1^,K_2_SO_4_ 0.70 mmol L^−1^,H_3_BO_3_ 0.01 mmol L^−1^,MnSO_4_·H_2_O 0.50 × 10^−3^ mmol L^−1^,ZnSO_4_·7H_2_O 0.50 × 10^−3^ mmol L^−1^,CuSO_4_·5H_2_O 0.20 × 10^−3^ mmol L^−1^,(NH_4_)_6_Mo_7_O_2_·4H_2_O 0.01 × 10^−3^ mmol L^−1^,Fe-EDTA 0.10 mmol L^−1^. Hydroponic culture vessels were positioned randomly in the growth chamber and rearranged once a week. The nutrient solutions were renewed every 5 days. Before Cu treatment, roots of all seedlings were blackened with activated charcoal and rinsed in deionized water to remove the excess powder. Seedlings were then treated with a range of Cu concentrations (0.25, 40, 160, and 320 μM), supplied as copper sulphate (CuSO_4_·5H_2_O). Here, 0.25 μM CuSO_4_ was the control. Because Cu is an essential plant micronutrient and our preliminary tests did suggest that 0.25 μM CuSO_4_ ensured good growth in all populations. Each treatment was repeated 6 times. After 25 days of Cu treatment, 6 seedlings were harvested from each treatment for Cu tolerance measurement. The presence of new roots (uncoated) visible beyond the charcoal-coated roots were recorded. The roots are in direct contact with the nutrient solution (or the soil solution in metalliferous soil under field conditions) and are therefore the first target for any toxic effect. Hence, the response of root growth is generally considered to be a very appropriate measure of the metal tolerance of plants. The determination of tolerance indices followed the methods described by Baker & Walker [45], and were calculated based on relative root growth (lengths) generated in parallel treatment and control units.

### Sampling strategy for populations used in AFLP and ISSR analyses

For AFLP and ISSR analyses, 12 and 13 populations of *R. acetosa * and *C. communis * were selected, respectively (Figure 1). For each population, at least 10 individuals were collected in the field at irregular intervals of 10–20 m in order to avoid collecting genetically-uniform individuals. Each population sampled covered a similar area of about 0.2 ha. All the plant materials were dried in silica gel before DNA extraction.

### DNA extraction

Genomic DNA was extracted using the cetyltrimethylammonium bromide (CTAB) protocol of Lodhi *et al. *[52] with minor modifications. Both the concentration and purity of the extracted DNA were checked using UV absorbance spectrophotometry. The resulting DNA samples were stored at −70°C and −20°C for long-term and short-term storage, respectively.

### AFLP protocol

AFLP analysis was carried out according to the method proposed by Vos *et al. *[53] with minor modifications. In brief, genomic DNA samples were digested using the enzyme combination *Eco *RI/*Mse *I and synthetic adaptors were ligated to the restriction fragments using T4 DNA ligase. A pre-amplification PCR was performed using AFLP primers having a single selective nucleotide (*EcoRI * + A/*MseI * + C). PCR products of the pre-amplification reaction were diluted 30-fold in sterile water then used as template for selective amplification using a combination of AFLP primers. In total, six selective primer pair combinations were used to generate fingerprints for the two species (Additional file 2: Table S6). Selective PCR products were then mixed with 2 μL of formamide loading dye. Mixtures were heated for 5 min at 94°C and then quickly cooled on ice. Three μL of each sample were loaded onto 6% denaturing polyacrylamide gel and run for 2 h at 50 W. Band patterns were visualized with a silver staining method [54]. The developed gel plate was then air-dried overnight and the images of AFLP profiles were recorded electronically with a scanner.

### ISSR protocol

Two DNA samples (one from the metallicolous population and the other from non-metallicolous population) from each species were selected to screen the ISSR polymorphisms by using 93 primers (UBC Set #9 plus all primers from the ISSR Resource Website http://www.biosci.ohio-state.edu/~awolfe/ISSR.html) in duplicate PCR reactions. Totally, 8 and 9 primers which produced highly readable, reproducible and polymorphic band patterns were selected for *R. acetosa * and *C. communis *, respectively (Additional file 2: Table S7). PCR amplification and gel electrophoresis analysis were carried out according to Yakimowski & Eckert [55].

### Chloroplast DNA sequencing

One individual from each sampled population was randomly selected for sequencing of two cpDNA regions (*psbJ-petA * and *3′rps16-5′trnK *). Primers and PCR protocols are as described in Shaw *et al. *[56]. PCR products were purified using High Pure PCR Product Purification Kit (Roche Diagnostics, USA) and were sequenced using an ABI 3730 sequencer. Both forward and reverse strands of *psbJ-petA * and *3′rps16-5′trnK * cpDNA regions were sequenced with a 100% overlap.

### Statistical and bioinformatic analysis

Data from hydroponic experiments were analyzed by using the statistical package SPSS 16.0 for Windows (SPSS Inc., USA) and non-parametric tests were used to assess the statistical significance of data.

Band patterns were scored in AFLP and ISSR fingerprint experiments and binary matrices for analysis were constructed for all populations of each species (POPGENE Version 1.32 [57]). Genetic diversity within populations was evaluated as the percentage of polymorphic loci, Nei’s gene diversity [58] and Shannon information index [59], using the software POPGENE Version 1.32 [57]. An independent-samples *t *-test as available in the SPSS statistical package was used to detect the significant differences in genetic diversity values between populations from cupriferous sites and those from uncontaminated sites. The same software was used to assess the correlation between the values of genetic diversity of each population and the associated Cu concentrations in soils. The partitioning of the genetic variance within and among populations and groups of populations was obtained with an analysis of molecular variance (AMOVA; [60]) conducted by Arlequin 3.5.1 [61]. For each species, two alternative groupings of populations were examined: (i) a geographically based arrangement of populations with sampling sites located in different provinces of China; and (ii) a strictly edaphic arrangement with Cu-contaminated sites vs. uncontaminated sites. The analyses of three hierarchical levels were conducted in order to partition the genetic variance into components attributable to different hierarchical levels, *i.e. * between groups, among populations, and within populations. The significance levels of variance components were calculated by 1000 permutations in each analysis. Dendrograms of the populations of the two metallophytes were constructed from Slatkin’s linearized pairwise *F *_*ST *_ calculated by Arlequin 3.5.1 [61], using the Neighbor-Joining method implemented in the program MEGA Version 4.1 [62]. Significance levels of pairwise *F *_*ST *_ were computed after 1000 random permutations Slatkin’s linearized pairwise *F *_*ST *_ values were compared with geographical distances between populations according to the Mantel’s test [63] with the implementation present by Arlequin 3.5.1 [61]; normalized Mantel Z-statistics were calculated after 1000 permutations.

Chloroplast DNA sequences were concatenated and then aligned using ClustalW algorithm resident within BioEdit (version 7.0.4.1; [64]). Haplotype networks were obtained on aligned sequences by NETWORK software ver. 4.6 (Fluxus Technologies Ltd.) by using the Reduced Median method.

## Authors’ contributions

MY designed experiments, carried out laboratory and statistical analysis, and drafted the manuscript. BL designed experiments and carried out the molecular marker studies. JTL, MH, WCL performed the analyses, AM constructed sequence alignment and helped to draft the manuscript. WSS designed and coordinated the project and drafted the manuscript. All authors discussed data and drafted the manuscript. All authors read and approved the final manuscript.

## Supplementary Material

Additional file 1 This file provides the tolerance indices for the 6 *Rumex acetosa * populations and 10 *Commelina communis * populations in the four CuSO_4_ treatments. Click here for file

Additional file 2 This file provides some supporting information for the article, including the result of the Mantel’s test, the results of Slatkin’s linearized pairwise FST, the results of AMOVA, GenBank accession numbers of two cpDNA non-coding regions, the lists of primers for AFLP and ISSR analysis. Click here for file
