# Supplementary material for: Contrasting patterns of genetic divergence in two sympatric pseudo-metallophytes: Rumex acetosa L. and Commelina communis L
Source: BMC Evol Biol. 2012 Jun 13;12:84. doi: 10.1186/1471-2148-12-84 (PMC3517898; doi:10.1186/1471-2148-12-84)
Supplement: Additional file 1 — This file provides the tolerance indices for the 6 Rumex acetosa populations and 10 Commelina communis populations in the four CuSO4 treatments. [file 1471-2148-12-84-S1.doc]

Growth ratio of root length (%)

Cu concentration (μM L^-1^) in solution

**Additional file 1_Figure S1.** The growth ratio of root length (%) of 6 *Rumex acetosa* populations under 4 Cu^2+^ treatments.

| **a**  Cu concentration (μM L^-1^) in solution  Growth ratio of dry weight (%) |
| --- |
| **b**  Cu concentration (μM L^-1^) in solution  Growth ratio of dry weight (%) |

**Additional file 1_Figure S2.** The growth ratio of root length (%) of 10 *Commelina communis* populations under 4 Cu^2+^ treatments. a: five contaminated populations; b: five uncontaminated populations.
