# Supplementary material for: Contrasting patterns of genetic divergence in two sympatric pseudo-metallophytes: Rumex acetosa L. and Commelina communis L
Source: BMC Evol Biol. 2012 Jun 13;12:84. doi: 10.1186/1471-2148-12-84 (PMC3517898; doi:10.1186/1471-2148-12-84)
Supplement: Additional file 2 — This file provides some supporting information for the article, including the result of the Mantel’s test, the results of Slatkin’s linearized pairwise FST, the results of AMOVA, GenBank accession numbers of two cpDNA non-coding regions, the lists of primers for AFLP and ISSR analysis. [file 1471-2148-12-84-S2.doc]

**Additional file 2_Table S1.** The Cu concentrations in shoots and roots of different *Rumex acetosa* and *Commelina communis* populations under four Cu^2+^ treatment in hydroponics (mg kg^-1^, DW; mean ± SD, n ≥3).

| Species | Populations | | 0.25 μmol L^-1^ | | 40 μmol L^-1^ | | 160 μmol L^-1^ | | 320 μmol L^-1^ | |
| --- | --- | --- | --- | --- | --- | --- | --- | --- | --- | --- |
|  |  |  | Shoot | Root | Shoot | Root | Shoot | Root | Shoot | Root |
| *Rumex acetosa* | | DGSCK(NM) | 4.2±0.7 ^a^ | 8.9±1.5 ^a^ | 231.7±76.9 ^a^ | 941.2±67.5 ^c^ | nd | nd | nd | Nd |
|  |  | CZ(M) | 9.2±2.9 ^ab^ | 8.9±1.9 ^a^ | 248.6±26.2 ^a^ | 780.4±15.2 ^b^ | 1495.8±353.7 ^b^ | 3236.5±336.6 ^c^ | 1046.3±144.1 ^a^ | 3558.3±305.6 ^bc^ |
|  |  | SZS(M) | 11.6±2.55 ^b^ | 21.3±6.3 ^c^ | 441.9±33.3 ^b^ | 789.9±115.0 ^b^ | 1148.8±183.5 ^ab^ | 2643.9±125.6 ^b^ | 1591.5±305.8 ^c^ | 3861.8.1±73.4 ^c^ |
|  |  | FHS(M) | 12.6±3.9 ^b^ | 17.3±3.3 ^bc^ | 303.8±60.5 ^a^ | 680.9±83.6 ^ab^ | 1445.2±158.8 ^b^ | 2618.8±99.5 ^b^ | 1333.6±106.9 ^b^ | 3291.5±200.5 ^b^ |
|  |  | TLCK(NM) | 8.3±1.6 ^ab^ | 11.2±3.2 ^ab^ | 201.5±36.4 ^a^ | 563.3±70.8 ^a^ | 875.3±210. 8 ^a^ | 1668.6±266.6 ^a^ | nd | Nd |
|  |  | DGS(M) | 12.3±1.9 ^b^ | 22.8±3.0 ^c^ | 394.4±48.6 ^b^ | 1001.8±52.1 ^c^ | 857.2±98.2 ^a^ | 1642.9±255.8 ^a^ | 1250.1±67.9 ^b^ | 2617.3.1±241.9 ^a^ |
| *Commelina communis* | | DGSCK(NM) | 21.6±2.5 ^a*^ | 88.9±16.6 ^a^ | 200.0±54.2 ^a^ | 868.5±108.4 ^ab^ | 419.7±14.2 ^b^ | 1841.5±34.1 ^b^ | nd | Nd |
|  |  | CZ(M) | 7.4±1.8 ^b^ | 28.4±3.7 ^b^ | 205.2±4.1 ^a^ | 1242.6±72.2 ^cd^ | 788.6±45.6 ^c^ | 2368.9±280.8 ^c^ | 1135.5±52.6 ^ab^ | 3332.8±143.9 ^a^ |
|  |  | SZS(M) | 4.9±0.1 ^b^ | 44.2±7.4 ^b^ | 226.3±12.7 ^a^ | 1416.0±173.7 ^d^ | 268.0±44.3 ^a^ | 2489.7±211.6 ^c^ | 851.2±27.0 ^a^ | 4344.6±48.1 ^b^ |
|  |  | FHS(M) | 7.6±1.7 ^b^ | 127.9±26.6 ^c^ | 277.2±35.6 ^c^ | 1149.2±133.6 ^bcd^ | 1050.8±62.2 ^d^ | 3228.2±85.9 ^e^ | 1329.8±129.8 ^b^ | 4557.7±213.9 ^b^ |
|  |  | TLCK(NM) | 21.7±2.6 ^a^ | 47.3±1.8 ^b^ | 365.8±37.9 ^d^ | 1040.8±97.6 ^abc^ | nd | nd | nd | Nd |
|  |  | DGS(M) | 6.5±2.2 ^b^ | 79.7±20.8 ^a^ | 308.5±24.4 ^c^ | 1058.1±61.4 ^abc^ | 503.1±8.0 ^b^ | 2936.5±122.2 ^d^ | 802.16±10.48 ^a^ | 5007.13±14.23 ^b^ |
|  |  | SCCK(NM) | 22.6±6.6 ^a^ | 88.9±11.9 ^a^ | 64.0±4.8 ^b^ | 792.7±104.4 ^a^ | nd | nd | nd | Nd |
|  |  | JSCK(NM) | 10.4±2.1 ^b^ | 95.9±5.8 ^a^ | 88.5±3.1 ^b^ | 1046.3±156.3 ^abc^ | 252.1±26.4 ^a^ | 1752.8±136.4 ^b^ | nd | Nd |
|  |  | AJS(M) | 3.0±0.9 ^b^ | 39.6±0.8 ^b^ | 216.5±31.1 ^a^ | 1161.0±133.0 ^bcd^ | 626.2±14.0 ^bc^ | 2593.2±21.9 ^c^ | 1454.5±90.1 ^b^ | 3168.1±80.7 ^a^ |
|  |  | CZCK(NM) | 6.5±2.3 ^b^ | 15.4±0.9 ^b^ | 85.7±21.6 ^b^ | 757.0±135.2 ^a^ | 346.5±22.3 ^ab^ | 1409.7±84.3 ^a^ | nd | Nd |

* Numbers followed by different letters in the same column within same species indicating a significant difference (P < 0.05);

**Additional file 2_Table S2.** Mantel’s test for correlation between geographical distances among populations and Slatkin’s linearized pairwise *F_ST_**

| Species | Marker type | r | P-value |
| --- | --- | --- | --- |
| *C. communis* | AFLP | 0.03 | 0.4 (n.s.) |
|  | ISSR | 0.39 | <0.005 |
| *R. acetosa* | AFLP | 0.19 | 0.1 (n.s.) |
|  | ISSR | 0.88 | <0.0001 |

* The plant species, marker type, correlation coefficient (r) and P-values after 1000 random permutations are reported. n.s. not significant

| **Additional file 2-Table S3.** Slatkin’s linearized pairwise *F_ST_*. Values between populations of *Commelina communis* and *Rumex acetosa*. | | | | | | | | | | | | | |
| --- | --- | --- | --- | --- | --- | --- | --- | --- | --- | --- | --- | --- | --- |
| AFLP *C. communis* | | |  |  |  |  |  |  |  |  |  |  |  |
|  | DGS | FHS | SZS | CZ | AJS | TLS | TSK | DGSCK | TLCK | CZCK | SCCK | JSCK | HZCK |
| DGS | 0.000 |  |  |  |  |  |  |  |  |  |  |  |  |
| FHS | 0.153 | 0.000 |  |  |  |  |  |  |  |  |  |  |  |
| SZS | 0.161 | 0.117 | 0.000 |  |  |  |  |  |  |  |  |  |  |
| CZ | 0.145 | 0.128 | 0.130 | 0.000 |  |  |  |  |  |  |  |  |  |
| AJS | 0.286 | 0.264 | 0.276 | 0.256 | 0.000 |  |  |  |  |  |  |  |  |
| TLS | 0.168 | 0.154 | 0.171 | 0.139 | 0.226 | 0.000 |  |  |  |  |  |  |  |
| TSK | 0.131 | 0.135 | 0.136 | 0.113 | 0.241 | 0.075 | 0.000 |  |  |  |  |  |  |
| DGSCK | 0.154 | 0.159 | 0.162 | 0.127 | 0.225 | 0.140 | 0.067 | 0.000 |  |  |  |  |  |
| TLCK | 0.178 | 0.188 | 0.193 | 0.164 | 0.287 | 0.176 | 0.108 | 0.035 | 0.000 |  |  |  |  |
| CZCK | 0.181 | 0.186 | 0.195 | 0.161 | 0.277 | 0.161 | 0.092 | 0.027 | 0.046 | 0.000 |  |  |  |
| SCCK | 0.199 | 0.186 | 0.185 | 0.168 | 0.263 | 0.194 | 0.122 | 0.032 | 0.062 | 0.062 | 0.000 |  |  |
| JSCK | 0.237 | 0.218 | 0.206 | 0.201 | 0.295 | 0.202 | 0.157 | 0.069 | 0.110 | 0.098 | 0.098 | 0.000 |  |
| HZCK | 0.187 | 0.187 | 0.174 | 0.165 | 0.267 | 0.182 | 0.118 | 0.028 | 0.006 | 0.047 | 0.062 | 0.094 | 0.000 |
| ISSR *C. communis* | | |  |  |  |  |  |  |  |  |  |  |  |
|  | DGS | FHS | SZS | CZ | AJS | TLS | TSK | DGSCK | TLCK | CZCK | SCCK | JSCK | HZCK |
| DGS | 0.000 |  |  |  |  |  |  |  |  |  |  |  |  |
| FHS | 0.000 | 0.000 |  |  |  |  |  |  |  |  |  |  |  |
| SZS | 0.000 | 0.000 | 0.000 |  |  |  |  |  |  |  |  |  |  |
| CZ | 0.000 | 0.000 | 0.000 | 0.000 |  |  |  |  |  |  |  |  |  |
| AJS | 0.000 | 0.011 | 0.022 | 0.000 | 0.000 |  |  |  |  |  |  |  |  |
| TLS | 0.020 | 0.064 | 0.054 | 0.024 | 0.030 | 0.000 |  |  |  |  |  |  |  |
| TSK | 0.015 | 0.060 | 0.042 | 0.014 | 0.019 | 0.000 | 0.000 |  |  |  |  |  |  |
| DGSCK | 0.019 | 0.077 | 0.070 | 0.022 | 0.018 | 0.109 | 0.082 | 0.000 |  |  |  |  |  |
| TLCK | 0.023 | 0.066 | 0.074 | 0.033 | 0.012 | 0.105 | 0.079 | 0.000 | 0.000 |  |  |  |  |
| CZCK | 0.015 | 0.067 | 0.064 | 0.025 | 0.015 | 0.100 | 0.073 | 0.000 | 0.000 | 0.000 |  |  |  |
| SCCK | 0.031 | 0.087 | 0.086 | 0.052 | 0.065 | 0.139 | 0.125 | 0.000 | 0.000 | 0.000 | 0.000 |  |  |
| JSCK | 0.008 | 0.057 | 0.049 | 0.027 | 0.043 | 0.098 | 0.082 | 0.000 | 0.000 | 0.000 | 0.000 | 0.000 |  |
| HZCK | 0.031 | 0.092 | 0.086 | 0.050 | 0.064 | 0.150 | 0.137 | 0.000 | 0.007 | 0.000 | 0.000 | 0.000 | 0.000 |
|  | |  |  |  |  |  |  |  |  |  |  |  |  |
| AFLP *R. acetosa* | |  |  |  |  |  |  |  |  |  |  |  |  |
|  | DGS | JHS | AJS | TJ | FHS | SZS | CZ | JSCK | SCCK | DGSCK | CZCK | TLCK |  |
| DGS | 0.000 |  |  |  |  |  |  |  |  |  |  |  |  |
| JHS | 0.080 | 0.000 |  |  |  |  |  |  |  |  |  |  |  |
| AJS | 0.000 | 0.109 | 0.000 |  |  |  |  |  |  |  |  |  |  |
| TJ | 0.046 | 0.000 | 0.103 | 0.000 |  |  |  |  |  |  |  |  |  |
| FHS | 0.070 | 0.071 | 0.000 | 0.101 | 0.000 |  |  |  |  |  |  |  |  |
| SZS | 0.112 | 0.115 | 0.137 | 0.000 | 0.091 | 0.000 |  |  |  |  |  |  |  |
| CZ | 0.103 | 0.098 | 0.139 | 0.056 | 0.000 | 0.047 | 0.000 |  |  |  |  |  |  |
| JSCK | 0.038 | 0.061 | 0.070 | 0.083 | 0.052 | 0.000 | 0.144 | 0.000 |  |  |  |  |  |
| SCCK | 0.107 | 0.155 | 0.074 | 0.192 | 0.229 | 0.130 | 0.000 | 0.244 | 0.000 |  |  |  |  |
| DGSCK | 0.199 | 0.232 | 0.216 | 0.275 | 0.282 | 0.201 | 0.141 | 0.000 | 0.058 | 0.000 |  |  |  |
| CZCK | 0.093 | 0.154 | 0.146 | 0.142 | 0.177 | 0.087 | 0.145 | 0.238 | 0.000 | 0.122 | 0.000 |  |  |
| TLCK | 0.100 | 0.134 | 0.137 | 0.153 | 0.171 | 0.051 | 0.100 | 0.223 | 0.071 | 0.000 | 0.145 | 0.000 |  |
| ISSR *R. acetosa* | |  |  |  |  |  |  |  |  |  |  |  |  |
|  | DGS | JHS | AJS | TJ | FHS | SZS | CZ | JSCK | SCCK | DGSCK | CZCK | TLCK |  |
| DGS | 0.000 |  |  |  |  |  |  |  |  |  |  |  |  |
| JHS | 0.007 | 0.000 |  |  |  |  |  |  |  |  |  |  |  |
| AJS | 0.006 | 0.000 | 0.000 |  |  |  |  |  |  |  |  |  |  |
| TJ | 0.000 | 0.000 | 0.000 | 0.000 |  |  |  |  |  |  |  |  |  |
| FHS | 0.000 | 0.017 | 0.016 | 0.009 | 0.000 |  |  |  |  |  |  |  |  |
| SZS | 0.000 | 0.018 | 0.015 | 0.007 | 0.000 | 0.000 |  |  |  |  |  |  |  |
| CZ | 0.000 | 0.040 | 0.037 | 0.035 | 0.000 | 0.000 | 0.000 |  |  |  |  |  |  |
| JSCK | 0.020 | 0.000 | 0.000 | 0.000 | 0.034 | 0.029 | 0.051 | 0.000 |  |  |  |  |  |
| SCCK | 0.020 | 0.048 | 0.036 | 0.052 | 0.010 | 0.015 | 0.000 | 0.039 | 0.000 |  |  |  |  |
| DGSCK | 0.000 | 0.005 | 0.008 | 0.000 | 0.000 | 0.000 | 0.000 | 0.014 | 0.031 | 0.000 |  |  |  |
| CZCK | 0.016 | 0.084 | 0.075 | 0.069 | 0.000 | 0.000 | 0.000 | 0.083 | 0.035 | 0.001 | 0.000 |  |  |
| TLCK | 0.000 | 0.017 | 0.023 | 0.013 | 0.000 | 0.000 | 0.000 | 0.014 | 0.023 | 0.000 | 0.000 | 0.000 |  |

*：Underlined values are not significant at P<0.05

**Additional file 2_Table S4.** Analysis of molecular variance (AMOVA) for *Rumex acetosa* and *Commelina communis* populations based on AFLP data (under two alternative grouping of populations: geographical and strictly edaphic).

| Source of variation | d.f. | Sum of squares | Variance | % of total |  | *P*-value |
| --- | --- | --- | --- | --- | --- | --- |
| *Rumex acetosa* | | | | | | |
| (a)Geographical partition (DGS, FHS, SZS, TS, DGSCK, TLCK, TSCK, SCCK vs. AJS, JHS, TJ, JSCK) | | | | | | |
| Between groups | 1 | 38.096 | 0.229 | 1.70 |  | < 0.01 |
| Among populations within groups | 10 | 258.988 | 1.407 | 10.45 |  | < 0.01 |
| Within populations | 108 | 1277.600 | 11.830 | 87.85 |  | < 0.01 |
| Total | 119 | 1574.683 | 13.465 |  |  |  |
| (b) Edaphic partition (DGS, FHS, SZS, TS, AJS, JHS, TJ vs. DGSCK, TLCK, TSCK, SCCK, JSCK) | | | | | | |
| Between groups | 1 | 56.140 | 0.549 | 4.04 |  | < 0.01 |
| Among populations within groups | 10 | 240.943 | 1.226 | 9.01 |  | < 0.01 |
| Within populations | 108 | 1277.600 | 11.830 | 86.95 |  | < 0.01 |
| Total | 119 | 1574.683 | 13.605 |  |  |  |
|  |  |  |  |  |  |  |
| *Commelina communis* | | | | | | |
| (a) Geographical partition (DGS, FHS, SZS, TS, DGSCK, TLCK, TSCK, SCCK vs. AJS, JSCK  vs. TLS, TSK vs. HZCK) | | | | | | |
| Between groups | 3 | 120.661 | 0.110 | 0.82 |  | 0.062 |
| Among populations within groups | 9 | 337.663 | 2.691 | 20.07 |  | < 0.001 |
| Within populations | 117 | 1241.000 | 10.607 | 79.11 |  | < 0.001 |
| Total | 129 | 1699.323 | 13.408 |  |  |  |
| (b) Edaphic partition (DGS, FHS, SZS, TS, AJS, TLS, TSK vs. DGSCK, TLCK, TSCK, SCCK, JSCK, HZ) | | | | | | |
| Between groups | 1 | 92.387 | 0.915 | 6.64 |  | < 0.001 |
| Among populations within groups | 11 | 365.936 | 2.266 | 16.43 |  | < 0.001 |
| Within populations | 117 | 1241.000 | 10.607 | 76.93 |  | < 0.001 |
| Total | 129 | 1699.323 | 13.788 |  |  |  |

**Additional file 2_Table S5.** Analysis of molecular variance (AMOVA) for *Rumex acetosa* and *Commelina communis* populations based on ISSR data (under two alternative grouping of populations: geographical and strictly edaphic).

| Source of variation | d.f. | Sum of squares | Variance | % of total |  | *P*-value |
| --- | --- | --- | --- | --- | --- | --- |
| *Rumex acetosa* | | | | | | |
| (a)Geographical partition (DGS, FHS, SZS, TS, DGSCK, TLCK, TSCK, SCCK vs. AJS, JHS, TJ, JSCK) | | | | | | |
| Between groups | 1 | 29.275 | 0.319 | 5.40 |  | < 0.01 |
| Among populations within groups | 10 | 37.658 | 0.878 | 15.18 |  | < 0.01 |
| Within populations | 168 | 938.400 | 4.586 | 79.30 |  | < 0.01 |
| Total | 179 | 1005.333 | 5.783 |  |  |  |
| (b) Edaphic partition (DGS, FHS, SZS, TS, AJS, JHS, TJ vs. DGSCK, TLCK, TSCK, SCCK, JSCK) | | | | | | |
| Between groups | 1 | 16.419 | 0.207 | 3.68 |  | < 0.01 |
| Among populations within groups | 10 | 58.248 | 1.108 | 19.71 |  | < 0.01 |
| Within populations | 168 | 930.667 | 4.306 | 76.61 |  | < 0.01 |
| Total | 179 | 1005.333 | 5.621 |  |  |  |
|  |  |  |  |  |  |  |
| *Commelina communis* | | | | | | |
| (a) Geographical partition (DGS, FHS, SZS, TS, DGSCK, TLCK, TSCK, SCCK vs. AJS, JSCK  vs. TLS, TSK vs. HZCK) | | | | | | |
| Between groups | 3 | 96.978 | 0.950 | 11.01 |  | < 0.01 |
| Among populations within groups | 9 | 117.925 | 2.099 | 24.33 |  | 0.07 |
| Within populations | 182 | 1437.600 | 5.584 | 64.69 |  | < 0.01 |
| Total | 194 | 1652.503 | 8.632 |  |  |  |
| (b) Edaphic partition (DGS, FHS, SZS, TS, AJS, TLS, TSK vs. DGSCK, TLCK, TSCK, SCCK, JSCK, HZ) | | | | | | |
| Between groups | 1 | 128.361 | 1.634 | 18.51 |  | < 0.01 |
| Among populations within groups | 11 | 96.541 | 0.911 | 10.32 |  | < 0.01 |
| Within populations | 182 | 1427.600 | 6.284 | 71.17 |  | < 0.01 |
| Total | 194 | 1652.503 | 8.829 |  |  |  |

**Additional file 2_Table S6.** GenBank accession numbers of two cpDNA non-coding regions (psbJ-petA and 3′rps16-5′trnK) of investigated *Rumex acetosa* and *Commelina communis* populations.

| Populations | *R. acetosa* | | *C. communis* | |
| --- | --- | --- | --- | --- |
|  | psbJ-petA | 3′rps16-5′trnK | psbJ-petA | 3′rps16-5′trnK |
| FHS | HM041054 | HM041071 | HM041095 | HM041083 |
| SZS | HM041063 | HM041077 | HM041091 | HM041090 |
| DGS | HM041060 | HM041074 | HM041097 | HM041086 |
| CZ | HM041065 | HM041066 | HM041101 | HM041078 |
| AJS | HM041057 | HM041068 | HM041092 | HM041080 |
| TLCK | HM041062 | HM041072 | HM041094 | HM041084 |
| SCCK | HM041058 | HM041069 | HM041099 | HM041081 |
| CZCK | HM041064 | HM041067 | HM041103 | HM041079 |
| DGSCK | HM041061 | HM041076 | HM041102 | HM041089 |
| JSCK | HM041055 | HM041070 | HM041093 | HM041082 |
| TJ | HM041056 | HM041073 | nd | nd |
| JHS | HM041059 | HM041075 | nd | nd |
| HZCK | nd | nd | HM041098 | HM041085 |
| TLS | nd | nd | HM041100 | HM041087 |
| TSK | nd | nd | HM041096 | HM041088 |

nd: no data available.

**Additional file 2_Table S7.**　The primers for AFLP analysis of *Rumex acetosa* and *Commelina communis.*

| *Rumex acetosa* | *Commelina communis* |
| --- | --- |
| E+AAT × M+CAA | E+AAG × M+CAT |
| E+AAC × M+CTT | E+ACG × M+CAT |
| E+AAC × M+CTC | E+AAC × M+CTC |
| E+AAC × M+CTA | E+ACT × M+CAG |
| E+ACT × M+CA | E+ATC × M+CTT |
| E+ACT × M+ CAC | E+ATC × M+ CTC |

**Additional file 2_Table S8.**　The primers for ISSR analysis in *Rumex acetosa* and *Commelina communis.*

| 8 primers for *Rumex acetosa* | | 9 primers for *Commelina communis* | |
| --- | --- | --- | --- |
| No. | Primer sequences | No. | Primer sequences |
| 818 | CAC ACA CAD ACA CAC AG | 808 | AGA GAG AGA GAG AGA GC |
| 819 | GTG TGT GTG TGT GTG TA | 809 | AGA GAG AGA GAG AGA GG |
| 836 | AGA GAG AGA GAG AGA GYA | 817 | CAC ACA CAC ACA CAC AA |
| 841 | GAG AGA GAG AGA GAG AYC | 823 | TCT CTC TCT CTC TCT CC |
| 873 | GAC AGA CAG ACA GAC A | 836 | AGA GAG AGA GAG AGA GYA |
| 880 | GGA GAG GAG AGG AGA | 864 | ATG ATG ATG ATG ATG ATG |
| 885 | BHB GAG AGA GAG AGA GA | 880 | GGA GAG GAG AGG AGA |
| 889 | DBD ACA CAC ACA CAC AC | 884 | HBH AGA GAG AGA GAG AG |
|  |  | 885 | BHB GAG AGA GAG AGA GA |
